# Propulsion-enhanced chemotaxis drives bifunctional enzymatic nanorobots deep into tumors

**DOI:** 10.1093/nsr/nwag022

**Published:** 2026-01-14

**Authors:** Suping Li, Guangjun Nie, Yuliang Zhao

**Affiliations:** National Center for Nanoscience and Technology of China, Chinese Academy of Sciences, China; National Center for Nanoscience and Technology of China, Chinese Academy of Sciences, China; Institute of Nanotechnology And Intelligence (inAI), and College of Chemistry and Materials Sciences, Jinan University, China; National Center for Nanoscience and Technology of China, Chinese Academy of Sciences, China

A central bottleneck in solid-tumor nanomedicine is physical access rather than molecular recognition; despite increasingly sophisticated targeting ligands, many nanocarriers still depend on passive circulation and stochastic extravasation across dysfunctional tumor vessels to reach tumor tissue [1]. Once in the bloodstream, these carriers are subject to dilution, vascular shear, interstitial resistance and Brownian motion, resulting in inefficient accumulation in poorly perfused and deeply embedded tumor niches [2]. In a recent *National Science Review* study, Yang and colleagues [3] shifted the emphasis from ‘better binding’ [4,5] to ‘better transport’, demonstrating that fully autonomous, chemically powered nanorobots can be engineered to actively navigate to tumors, yielding pronounced therapeutic gains. Importantly, the work reframes chemotaxis from a qualitative phenomenon into a quantitatively designable mode of *in vivo* transport, with implications that extend beyond a single material system.

To navigate effectively, a nanorobot must generate sufficient propulsive force to overcome translational diffusion, and simultaneously sufficient orientation torque to resist rotational Brownian randomization and maintain alignment with a chemical gradient. Most chemically powered swimmers can achieve high speeds but drift directionally, whereas gradient-sensing designs often exhibit biases too weak to overcome thermal noise arising from incessant molecular agitation in physiological environments. Yang and colleagues [3] formalized a unifying concept, namely propulsion-enhanced chemotaxis, in which strong propulsion not only increases displacement but also stabilizes directional bias by allowing chemotactic torque to dominate stochastic reorientation. This framing is powerful because it renders chemotaxis an engineerable balance of forces and torques, rather than a descriptive label inferred from trajectories (Fig. 1).

**Figure 1.**
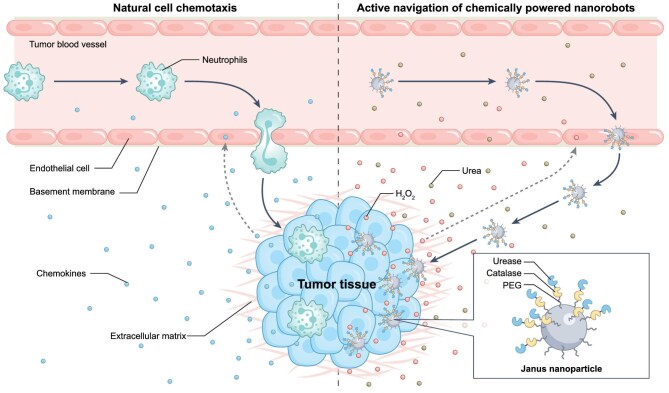
Natural versus synthetic chemotaxis. Left: neutrophils exhibit biological chemotaxis by sensing tumor-derived chemokines (e.g. CCL2 and CCL5), enabling them to extravasate across breast-tumor vascular barriers and overcome high interstitial pressure to infiltrate the tumor. Right: chemically powered nanorobots use urea as a propellant while steering along tumor-specific hydrogen peroxide gradients, thereby actively navigating and penetrating deep into tumor tissue.

To realize this principle, the authors designed bifunctional enzyme-powered Janus nanorobots that integrate propulsion and steering within a single nanoscale architecture [3]. By asymmetrically organizing urease and catalase on a Janus scaffold, the system exploits tumor-relevant chemical cues to generate both sustained thrust and gradient-aligned orientation. Urease provides continuous propulsion using chemical ‘fuel’ that is abundant in the blood and tumor microenvironment, whereas catalase responds to pathological signals, such as elevated hydrogen peroxide, to bias orientation. The value of the bifunctional enzyme strategy lies not in additive catalysis but in behavioral integration. By using a high-concentration background fuel (urea) for energy conversion and a low-concentration specific signal (hydrogen peroxide) for directional control, the system achieves a coherent migration phenotype that persists in the presence of physiological noise. Conceptually, this approach proposes a general strategy for programmable navigation by matching disease-specific fuels and signals with asymmetric catalytic layouts, thereby enabling chemotaxis to be tuned to distinct microenvironmental landscapes. The *in vivo* consequences reported are striking in magnitude and scope. Following intravenous administration in breast tumor-bearing mice, the bifunctional enzyme nanorobots achieve a 209-fold increase in tumor-targeting delivery efficiency (Ɛ) relative to passive counterparts. This enhanced arrival is accompanied by a greater than 10-fold increase in tumor penetration depth and an extraordinary 1970-fold enhancement in cellular uptake. These metrics correspond to three traditionally coupled bottlenecks—vascular escape, interstitial transport and cell entry—that are rarely improved simultaneously. Their concurrent amplification indicates that active, biased migration has begun to dominate steps typically limited by diffusion. Therapeutically, when loaded with the photosensitizer Ce6 for photodynamic therapy, the chemotactic nanorobots achieve ∼92.7% tumor growth inhibition, corresponding to an overall ∼49-fold improvement in antitumor efficacy relative to passive delivery. Notably, the authors emphasize that comparable therapeutic outcomes can be achieved with drug doses reduced to ∼1% of those required by conventional nanocarriers, highlighting chemotaxis as a potential route to decouple efficacy from dose escalation.

This work naturally complements parallel advances in biohybrid microrobotics, exemplified by enzyme-powered Janus platelet cell robots [6], which emphasize biomimetic interfaces, immune compatibility and active targeting. While such cell-based systems excel in biological integration, the present study advances a more explicitly computable navigation paradigm, in which transport efficiency is governed by the measurable interplay between propulsion and orientation. These two trajectories are not competing, but likely convergent; future platforms may combine biomimetic or cell-derived exteriors for circulation stability with multimodule propulsion–steering units for deep-tissue access and directional control. Several challenges will determine the clinical generality of ultrasensitive chemotaxis: (i) the realism and heterogeneity of chemical gradients across tumor types, stages and treatment contexts; (ii) the stability and failure modes of propulsion and torque generation under protein corona formation, flow and enzyme drift; and (iii) system-level optimization in combination regimens, where spatial distribution and timing rather than dose alone govern synergy with photodynamic, immunogenic or radiosensitizing therapies. By articulating chemotaxis as a force- and torque-balanced transport mode and demonstrating order-of-magnitude gains in delivery and efficacy *in vivo*, this *NSR* study argues convincingly that propulsion-enhanced chemotaxis can evolve from an experimental curiosity into a programmable tool for precision cancer therapy.

## References

[1] Sindhwani S, Syed AM, Ngai J et al. Nat Mater 2020; 19: 566–75. 10.1038/s41563-019-0566-2 31932672

[2] Ouyang B, Poon W, Zhang YN et al. Nat Mater 2020; 19: 1362–71. 10.1038/s41563-020-0755-z 32778816

[3] Yang ZL, Pei ZY, Gao ZX et al. Natl Sci Rev 2026; 13: nwaf580. 10.1093/nsr/nwaf580 41640638 PMC12866669

[4] Li SP, Jiang Q, Liu SL et al. Nat Biotechnol 2018; 36: 258–64. 10.1038/nbt.4071 29431737

[5] Li SP, Zhang YL, Ho SH et al. Nat Biomed Eng 2020; 4: 732–42. 10.1038/s41551-020-0573-2 32572197

[6] Tang SS, Zhang FY, Gong H et al. Sci Robot 2020; 5: eaba6137. 10.1126/scirobotics.aba6137 33022613

